# Integrating Patient-Centred Research in the Canadian Cancer Trials Group

**DOI:** 10.3390/curroncol28010062

**Published:** 2021-01-21

**Authors:** J. Needham, J. Taylor, D. Nomikos

**Affiliations:** Canadian Cancer Trials Group, Queen’s University, Kingston, ON K7L 3N6, Canada; jtaylor@ctg.queensu.ca (J.T.); dnomikos@ctg.queensu.ca (D.N.)

**Keywords:** cancer, patient and public engagement, stakeholder engagement, patient-centred research, patient representatives, clinical trials

## Abstract

The inclusion of patients as partners in research is a key link in the delivery of patient-centred care in healthcare systems. Despite genuine intentions to engage patients in authentic partnerships, efforts can result in tokenism and benefits of engagement are missed. Understanding how patient engagement provides value along the research to patient-care continuum and how to best engage patients as partners are key. This document describes the method taken by the Canadian Cancer Trials Group (CCTG) to implement meaningful patient centricity and engagement and the benefits realized. Originally, Patient Representatives were recruited and assigned to CCTG Committees. Lacking guidance, the role was one of a passive meeting attendee. A gap analysis identified a need for clarity in expectations, understanding of the linkage to CCTG strategic objectives, and supporting tools and training. A plan was developed and successfully implemented in three phases, each phase building on the previous, the level of patient engagement simultaneously changing from “Inform” to “Involve” to “Collaborate” on the International Association for Public Participation (IAP2) scale. Results include significant contributions to increased patient accrual in CCTG trials, to increased CCTG grant funding, as well as recognition and adoption of these practices within Canada and internationally.

## 1. Introduction

**Figure 1:**
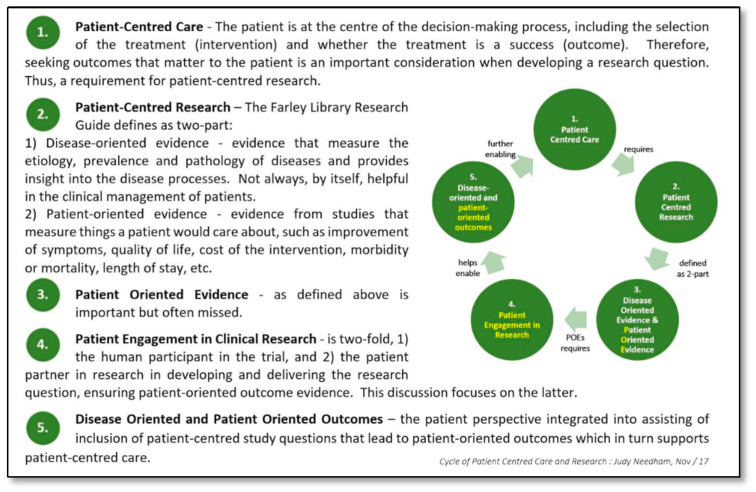
The Value of Patient Engagement in Research.

Establishing partnerships with patients to engage them in all levels of development and execution of clinical trials (CTs) is increasingly being explored and applied globally [1,2,3,4,5,6]. Including the patient perspective when designing research studies or setting the research agenda is new to the Canadian research landscape compared to frameworks such as the Patient-Centered Outcomes Research Institute (PCORI) and the Clinical Trials Transformation Initiative (CTTI) in the US or INVOLVE (UK national advisory group) in the UK [7,8,9]. Advantages of involving the end-user groups in the broad research agenda can include end-user priorities and perspectives being considered in setting research priorities, developing proposals, recruitment and retention of patients, and disseminating results. Importantly, these benefits can feed back to the end user in the form of societal benefits of patient-centred care (Figure 1). Although recommendations and guidelines are emerging on research partnerships with patients, literature on methods of implementing these partnerships is limited. 

The CCTG was established in 1980 and is a cooperative academic oncology group that designs and administers Phase I–III clinical trials across all cancer types in cancer therapy, supportive care, and prevention across Canada through a collaborative network of researchers, physicians, scientists, statisticians, and patient partners. The CCTG also collaborates with research groups and centres in the United States, Europe and elsewhere and is internationally recognized for conducting practice changing clinical trials leading to new treatments that give people with cancer longer, better-quality lives.

The CCTG has led the way in Canada in developing a meaningful patient engagement model and including the patient perspective as it sets and executes its research agenda. This is evidenced by positive impacts in setting research priorities, better recruitment and retention of patients, and by numerous requests from organizations both nationally and internationally for access to the methodology, training materials, and tools that have been developed, including the Canadian Cancer Research Alliance, Ontario Institute for Cancer Research, Colorectal Cancer Canada, Myeloma Canada, Ovarian Cancer Canada, and the National Cancer Institute (NCI) National Cancer Trials Network (NCTN) partner groups.

## 2. Materials and Methods

**Figure 2:**
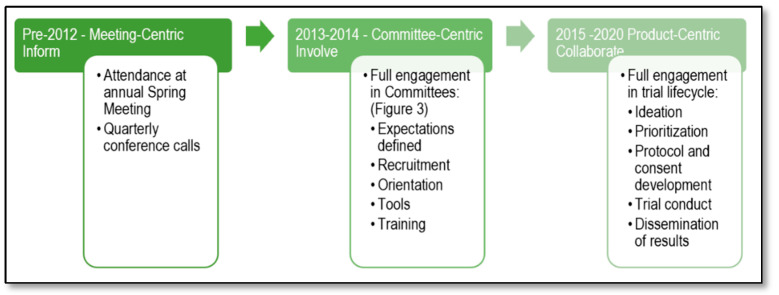
Phases of Patient Representative Engagement Implementation in the Canadian Cancer Trials Group.

CCTG recognized the importance of integrating the patient perspective into clinical trial development and execution. The process involved moving through three phases in developing and implementing its patient engagement model (Figure 2).

### 2.1. Phase I: Pre 2012 Meeting-Centric—Inform Level of Engagement

**Figure 3:**
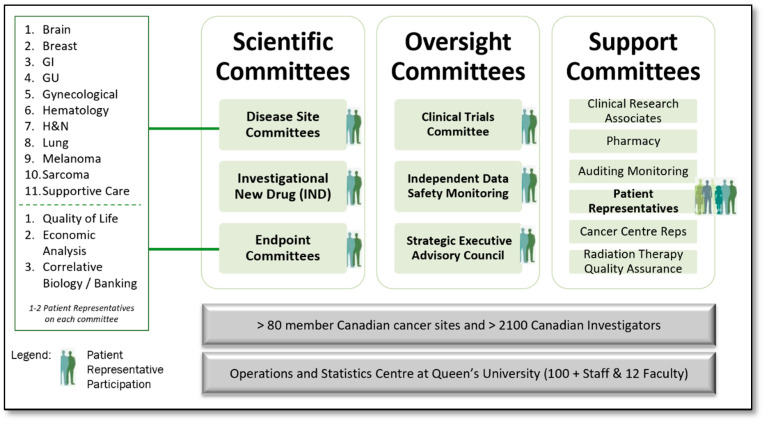
Patient Engagement in the Canadian Cancer Trials Group Committee Structure.

A standard methodology for approaching patient engagement in research organizations is often limited to inviting patients to attend conferences and meetings (meeting-centric). CCTG Patient Representatives were and still are recruited and assigned to each of the eleven Disease Site Scientific Committees (DSCs), the Investigational New Drug (IND), Quality of Life, Economic Analysis, and the Correlative Science Tumour Banking (CSTB) Committees. At a governance level, Patient Representatives are members of the Clinical Trials Committee (CTC) that prioritizes and approves new trial proposals for further development, and the Strategic Executive Advisory Council (SEAC). Patient Representatives are also members of the independent Data Safety Monitoring Committee (DSMC). Collectively, the Patient Representatives constitute their own committee, the Patient Representative Committee (Figure 3).

Although recruited and assigned to committees, the CCTG Patient Representative role was also limited to one of a passive meeting attendee, primarily at the Annual Spring Meeting of Participants and secondly at periodic committee conference calls, with the objective of trying to provide the patient perspective to the best of one’s ability. Lacking training and guidance, the role was at a basic inform (IAP2) level, often resulting in a feeling of non-contributing tokenism on the part of the Patient Representative, and no measurable contributions to patient-centred research on the part of the committees or the CCTG. 

CCTG recognized the need for further structure, role definition, and guidance.

### 2.2. Phase II: Meeting-Centric to Committee-Centric—Involve Level of Engagement

A report prepared by a Patient Representative (Needham, J., Advancing the Role of the Lay Representative in NCIC CTG (Canadian Cancer Trials Group, Kingston, ON, Canada) Personal Communication, 2013) [10] subsequent to attending the 2013 CCTG Annual Spring Meeting of Participants discussed the level of engagement experienced (informative) versus what a potential target level of engagement (collaborative) could be. The report included a proposed implementation plan to bridge the gaps which assisted in formulating the next steps. A literature search including reviews of INVOLVE, PCORI, CTTI, and the IAP2 [11] frameworks affirmed the pathway. The report, or gap analysis, provided the methodology to build the path.

**Figure 4:**
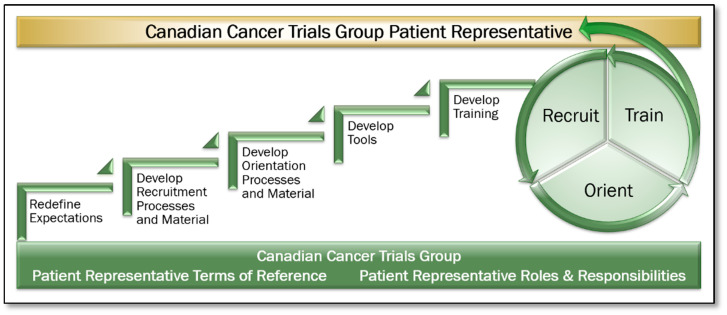
Elevating the Canadian Cancer Trials Group Patient Representative Role.

Five areas were identified and addressed, as indicated in Figure 4:

1. *Expectations:* Patient Representative Terms of Reference and Roles and Responsibilities documents were created and approved. Expectations of Patient Representative participation within their committees were defined, including accessing electronic trial, committee, and meeting information, how to prepare for committee meetings and the time commitments required.
2. *Recruitment:* A formal recruitment process including definition of desired role attributes, a position posting, an interview guide, and associated processes were developed. The volunteer opportunities were distributed to Clinical Trial Units, Investigators, the Canadian Cancer Society, and other organizations throughout Canada. Applicants were interviewed and selected through a panel interview process. The Patient Representative term is a once renewable three-year term.
3. *Orientation:* An orientation process was developed to provide introduction to the organization, leadership, key contacts, and an introduction to a progressive training program.
4. *Tool Development:* Tools including a Patient Representative Manual, Portfolio Trial Summary Matrix Report and Trial Tracking Forms were developed. The Portfolio Trial Summary Matrix provides a snapshot of all trials in a disease site at a given point in time, including where each is at in its lifecycle, including current accrual status. The Trial Tracking Form enables the Patient Representative to identify all key aspects of the trial, and secondly, to record and track patient feasibility, accrual related issues, and other notes on a cumulative basis, enabling discussion of accrual related issues and outcomes within their respective committees. This also enabled a continuum from meeting to meeting.
5. *Education and Training:* Training materials including the Patient Representative Manual and in-person or virtual training module were developed. Initial Patient Representative training was conducted in person at the CCTG Annual Spring Meeting of Participants. Ensuring all interdisciplinary committee members are supportive of and engaged in the process to enhance Patient Representatives’ roles within their committees was paramount. All onboarding methods and procedures, training materials, and tools were shared with committees in person also at the CCTG Annual Spring Meeting of Participants. The same materials were shared with the investigative community and staff in the CCTG Central Operations and Statistics Centre, including Study Coordinators (project and data managers on CCTG clinical trials) who are well positioned to support the Patient Representatives in their roles. Refresher training and awareness of the CCTG patient engagement model is provided based on member attrition and turnover.

Using the Canadian Institute of Health Research (CIHR) Strategy for Patient-Oriented Research (SPOR) definition of patient [12,13] as guidance, the core requirements for the Patient Representative role were defined primarily as having a personal experience with cancer or as a cancer patient caregiver. However, in recruiting, an effort was also made to achieve a pan-Canadian geographic patient representation. This provided a reasonable representation of broader Canadian patient communities. Recognizing that patients are people first [14], an unexpected benefit of diversity in professional backgrounds and other personal experiences brought a positive variety of perspectives to augment the scientific expertise at the committee tables. Broader diversity options will be explored as the model continues to grow and mature.

### 2.3. Phase III: Committee-Centric to Product-Centric—Collaborate Level of Engagement

**Figure 5:**
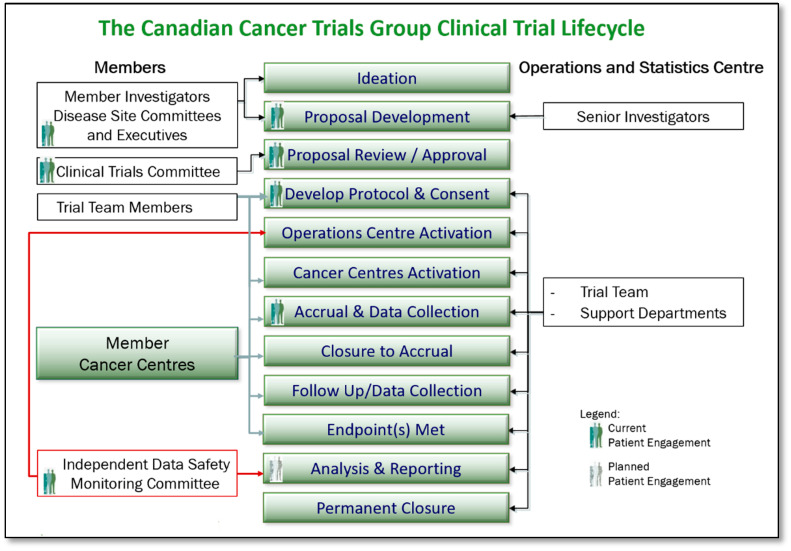
Patient Engagement in The Canadian Cancer Trials Group Clinical Trial Lifecycle.

Building on these successes, in 2015, a product (clinical trial)-centric model was initiated to add yet another layer of depth and further integrate the patient perspective into every step of the CCTG trial lifecycle, including concept proposal, design, approval, protocol, and consent development, accrual to and reporting of individual trials (Figure 5). The aim was to provide input in new proposals specific to patient-oriented end points, to patient feasibility including barriers to accrual, and to partner in raising awareness of specific trials and clinical trials in general. 

This approach is supported by research conducted by Ennis and Wykes in the UK [15], who found that studies involving patients were more likely to achieve recruitment targets due to: (1) the language used in materials being more appealing or easier to understand for patients because of vetting by other patients; (2) patients being able to contribute insight into the realities of living with a health problem and therefore understanding better which designs will be the least burdensome; and (3) patients being more willing to participate in research that they know has involved other patients.

Additionally, an analysis of the Canadian Institute of Health Research (CIHR) Strategy for Patient Oriented Research (SPOR) principles [12,13] of patient engagement and models in other countries (United Kingdom [4] and the United States [5]) confirmed the direction of adding this further depth to the role of Patient Representatives in order to influence clinical trial conduct.

The processes outlined below were implemented first by development of the additional forms and training modules for each of the identified touch points. The first Patient Representative training was again introduced in a face-to-face environment for both Patient Representatives and Disease Site Committee Members at a CCTG Annual Spring Meeting of Participants. Introduction sessions of materials and training for the appropriate CCTG Operations and Statistics Centre and Trial Team staff were also held. Refinement of tools (forms developed to facilitate Patient Representative input) was facilitated through user feedback. On-going training and procedures have been built into the Patient Representative Manual, training modules, and CCTG Operations and Statistics Centre new staff on-boarding materials.

As illustrated in Figure 5, the following processes were developed and implemented in order to enable patient engagement/formal Patient Representative input in the CCTG clinical trial lifecycle:

1. *Ideation and Proposal Development*: Patient Representatives’ roles on CCTG Disease Site Committee Executives facilitate the ability to contribute to trial design, to inclusion of patient-centred end points, and to decisions regarding which proposals are sufficiently meritorious to be presented to the Clinical Trials Committee (CTC) for approval. A Patient Representative New Proposal Review form has been developed as a tool to facilitate the review and input.
2. *Trial Proposal Review and Approval*: As full members of the CTC, Patient Representatives review and score proposals that come forward for consideration. Those proposals that are approved by the CTC move on to become fully developed and activated trials conducted by the CCTG. Thus, Patient Representatives on the CTC are directly impactful on those trials that are made available to patients. Tools that support the Patient Representative in their role on the CTC are the same as afforded to all members of the committee, including CTC review, and scoring guidelines and a CTC New Proposal Review Form.
3. *Protocol and Consent Development*: As protocols and consents for CCTG trials are developed and finalized, Disease Site Committee Patient Representatives review, provide input, and make suggestions to help ensure the trial design is acceptable to patients. A Patient Representative Protocol Review Form and Consent Review Form have been developed to assist Patient Representatives.
4. *Trial Activation and Launch*: Patient Representatives have contributed to the development of a plain language template and now contribute to the development of plain language patient-facing materials (Research Ethics Board approved) for all CCTG trials. These are distributed to regional cancer centres to assist in raising awareness of clinical trials at Canadian centres as applicable, with the goals of assisting patient understanding and thereby influencing accrual.
5. *Trial Accrual and Progression Monitoring*: As members of the Disease Site Committee Executives, CCTG Disease Site Committee Patient Representatives are able to monitor trial progress within their portfolios and contribute to discussions around those trials that are having challenges. Tools including the Portfolio Trial Matrix Report, the Trial Tracking Form, Monthly Accrual Reports, and the online Accrual Tracking Utility assist Patient Representatives in this role. Additionally, as equal members of the Data Safety Monitoring Committee, Patient Representatives are aware of and contribute on each trial’s status, including trial progress and safety.
6. *Trial Closure, Analysis, and Reporting*: A planned next step in the evolution of the CCTG patient engagement model includes development of processes for informing knowledge translation associated with this research, and methods of supporting widespread dissemination of CCTG study findings across appropriate patient communities in Canada.

Monitoring and control checkpoints start with New Proposal reviews residing with the Clinical Trials Committee (CTC) Patient Representative members (Figure 3) as a checkpoint that all new proposals have been reviewed prior to CTC meetings. Monitoring and control of the Protocol, Consent, and Plain Language Summary reviews reside with the CCTG Study Coordinator as part of their interactions with the Patient Representatives and their trial development checklist. Overall results of all Patient Representative engagement throughout the clinical trial lifecycle are tracked by the Patient Representative Committee Central Office Support Individual and the Patient Representative Committee Chair and reported quarterly through the annual Operations Objectives Report.

## 3. Results

The CCTG Patient Representative and the collective Patient Representative Committee now display the capability and the potential to influence trial design and accrual and have demonstrated the following results:

### 3.1. CCTG Experiencing Trial Activity and Accrual at its Highest in Six Years: Qualitative Evidence of Patient Representatives Having Contributed to this Success

- 100% of CCTG trials are reviewed at key touchpoints as identified earlier, including new proposal, funding review, protocol, and consent development, launch and execution for inclusion of patient-centred end points, potential patient barriers to accrual and patient safety.
- The inclusion of patient-centred end points to augment the scientific question, and proactive review and identification of patient barriers in CCTG trials has contributed to increased trial enrollment. In addition to informing practice, patient-centred end points inform patients by providing outcomes that are important to them and their quality of life, thereby motivating enrollment and retention. This is related to a broader result of also making a difference to Canadian patients.
- An appeal influencing industry to reverse a decision to terminate drug supply for CCTG trial PA6 enabled trial continuation versus trial closure (Needham, J. Letter to Meryem Maoui, June 2015. Maoui, M. Letter to Judy Needham, July 2015).
- An appeal influencing United Kingdom participation in CO21 influencing broader global accrual (Needham, J. Letter to Vicky Coyle, Centre for Cancer Research and Cell Biology, Queen’s University Belfast, August 2013).
- Influencing new trials in the pipeline–MYX.1 A Phase II Study of High Dose Weekly Carfilzomib plus Cyclophosphamide and Dexamethasone in the Treatment of Relapsed Multiple Myeloma; coordinated through Mr. Aldo Del Col, CCTG Patient Representative on the Hematology DSC and then Chief Scientific Advisory of Myeloma Canada (1917).

### 3.2. CCTG has Realized a 50% Increase in Research Funding Realized in the Past Four Years: Patient Representative Committee Contributions to These Results

- The Patient Representative Committee scored high in the core programmatic grant application from the Canadian Cancer Society Research (Presentation; 2016 CCSRI Grant Review), thereby influencing the overall CCTG score. Committee structure and plans were well received with no changes requested.
- Evidence of patient engagement has become a criteria for many granting organizations. CCTG grant applications (CIHR, PCORI, etc.) now clearly articulate all patient representative touchpoints and engagement including specific associated activities throughout the lifecycle of the specific project and the trial team resulting in higher scores versus criticism and loss of points for lack of meaningful patient engagement.

### 3.3. Evidence of a Productive Well-Working Model and Elimination of Duplication of Effort Regarding Best Practices in Patient Engagement

- Internally
  - Patient Representatives are highly sought out for secondary responsibilities in addition to their primary duties outlined in this publication, not limited to, but including, activities such as cross-committee projects and working groups, grant applications, trial teams, and molecular tumour board membership.
- Externally
  - CCTG’s patient engagement model has been sought out by and shared with the Canadian Cancer Clinical Trials Network, Myeloma Canada, British Columbia Cancer, the Ontario Institute of Cancer Research, the Canadian Initiative for Patient Advocacy Groups in Clinical Trials, and National Cancer Institute (NCI) National Clinical Trials Network (NCTN) partner groups.
  - CCTG Patient Representatives have been requested to and support international committees including the NCI NCTN Accrual Core Team, Correlative Science Tumour Biology Committee, Patient Advocacy Chairs’ Committee, Social Media Workshop, Bethesda, MD, and NCI NCTN Plain Language Committee.
  - CCTG Patient Representatives are highly sought out for external to CCTG grant applications, presentations at meetings, publications, supporting national and international committees addressing patient engagement in clinical trials and reviews [16,17,18,19,20,21].

## 4. Discussion

Many research papers evaluating patient engagement indicate that patient engagement in healthcare research is likely feasible; however, it runs the risk of easily becoming tokenistic, and further identifies that research dedicated to identifying best methods to achieve meaningful engagement is lacking and clearly needed [22]. Through investment of time and resources, the CCTG has developed a method of meaningfully engaging and measuring the effectiveness of patients as partners as a strategic enabler for delivering patient-centred trials for Canadians. 

This CCTG framework differs from others in that it takes engagement beyond most other patient engagement in research frameworks that stop at recruiting Patient Representatives to committees with committee Terms of Reference as guidance, limiting engagement to an IAP2 “Inform” level of engagement. This model deliberately stepped the level of engagement up to the IAP2 “Involve” level in Phase II, then to the IAP2 “Collaborate” level in Phase III.

Individually, the role of the Patient Representative is now one of a collaborative strategic partner in the development and delivery of CCTG patient-centred trials. The CCTG has supported development of an in-depth role of its Patient Representatives that goes beyond filling a seat on a committee; it has identified and developed the touchpoints throughout the clinical trial lifecycle that would benefit from patient input, and has thereby truly integrated the patient perspective into all aspects of the clinical trial lifecycle. Patient Representative participation within strategic and oversight committees will continue to ensure that the patient perspective is present at leadership and oversight levels of the organization in terms of strategic planning, trial review and approval, independent data safety monitoring, and economic analysis.

Collectively as a committee unto itself, the Patient Representative Committee will continue to support achievement of CCTG’s strategic priorities by leveraging community connections to influence accrual to CCTG trials, developing partnerships with national and community organizations, developing outreach strategies for trial awareness and knowledge transfer of results, and utilizing the collective “patient voice” to present a pan-Canadian patient perspective to government and industry.

The development and implementation of guidance materials, processes, and tools helped facilitate a cultural shift to include patient voices and redefine partnerships between researchers and public participants within CCTG committees.

Immediate future perspectives for CCTG include the development and implementation of additional metrics to measure the impact of this patient engagement model, processes for informing knowledge translation associated with this research, and methods of supporting widespread dissemination of CCTG study findings across patient communities in Canada.

## 5. Conclusions

The CCTG has developed a patient engagement model that reflects a collaborative level of engagement and has shown qualitative, tangible benefits and results. The CCTG will continue to sustain this model, to develop it further to include Canadian Patient Advocacy Group voices, to explore further development of measurements of success, and will continue to aim to eliminate duplication of guidance through sharing practices and provide support for patient involvement in Canada and internationally. The CCTG Patient Representative model provides unique opportunities for other organizations who may wish to adopt similar models.
